# The effects of physical activity on overall survival among advanced cancer patients: a systematic review and meta-analysis

**DOI:** 10.1186/s12885-021-07988-1

**Published:** 2021-03-07

**Authors:** Naomi Takemura, Siu Ling Chan, Robert Smith, Denise Shuk Ting Cheung, Chia-Chin Lin

**Affiliations:** 1 School of Nursing, Li Ka Shing Faculty of Medicine, The University of Hong Kong, 4/F, William M.W. Mong Block, 21 Sassoon Road, Pokfulam, Hong Kong; 2 School of Nursing, College of Nursing, Taipei Medical University, Taipei City, Taiwan; 3 Alice Ho Miu Ling Nethersole Charity Foundation Professor in Nursing, Pokfulam, Hong Kong

**Keywords:** Advanced cancer, Meta-analysis, Physical activity, Survival

## Abstract

**Background:**

The survival rates of advanced cancer patients remain low despite clinical therapy advancements. However, physical activity showed promising effects in improving cancer outcomes. This review aimed to systematically evaluate and synthesize the effects on overall mortality of post-diagnosis physical activity in advanced cancer patients.

**Methods:**

A systematic search of six English databases (PubMed, EMBASE, CINAHL, PsycINFO, The Cochrane Central Register of Controlled Trials, and SPORTDiscus) was conducted from their inception up to 3 February 2021. The association of physical activity with survival was evaluated by combining study-specific hazard ratios with random-effects meta-analysis models.

**Results:**

Eleven studies were identified. Compared with the reference group, higher-level physical activity was not significantly associated with a lower risk of earlier mortality in advanced cancer patients (InHR = − 0.18, 95% CI, − 0.36 to 0.01). When separated by study type, a higher level of physical activity in non-randomised trials was significantly associated with reduced mortality risk (InHR = − 0.25, 95% CI: − 0.44, − 0.06). However, in randomised trials, engaging in exercise was not significantly associated with a lower mortality risk compared with the control group (InHR = 0.08, 95%CI: − 0.17, 0.32).

**Conclusions:**

Discrepancies were uncovered in the effect of physical activity on overall survival in randomised and non-randomised trials. In non-randomised trials, a higher level of physical activity was significantly associated with a lower risk of mortality, whereas no significant effect on survival was observed during exercise interventions compared to the control in randomised trials. Considering the wider benefits of physical activity, exercise can still be recommended to improve outcomes for advanced cancer patients. Nevertheless, it might be too late for advanced cancer patients to start exercising for survival improvements, based on findings from randomised controlled trials.

**Supplementary Information:**

The online version contains supplementary material available at 10.1186/s12885-021-07988-1.

## Background

In 2018, there were 18 million new cases of cancer worldwide, the second leading cause of death (estimated at 9.6 million) [1]. The most common newly diagnosed cancers were lung, breast, and colorectal cancers [1]. Among these, approximately half of the newly diagnosed lung and colorectal cancer patients are at an advanced or metastatic stage. Moreover, the majority of patients diagnosed at an earlier stage of the disease eventually develop tumour progression [2, 3]. The 5-year relative survival rates for numerous advanced-stage cancers such as lung, colorectal, breast, liver, and pancreatic remain low, ranging from 2 to 27% [4]. Ultimately, patients with advanced cancer are susceptible to substantial physical and psychological distress that exacerbate near the end of life [5].

Physical activity is being progressively studied as a nonpharmacologic intervention to maximise health benefits and outcomes in a healthy general population and in populations with chronic illnesses and cancers [6, 7]. Along with improving physical and psychological well-being, engaging in regular physical activity or increasing levels of physical activity were associated with decreased mortality risk and longer healthy living among healthy adults and elderly populations [8–11]. Some reviews have even reported an association between pre- and post-diagnosis physical activity and cancer survival. A review of prospective cohort studies in cancer patients at all stages suggested that higher levels of physical activity decreased the risk of cancer-related mortality, specifically in breast, and colorectal cancer populations [12]. Furthermore, findings from a recent large-scale meta-analysis that included both randomised trials and cohort studies reported that physical activity added to survival benefits in prostate, lung, liver, stomach, oesophageal, and female reproductive cancers of various stages [13]. Potential biological mechanisms have been proposed to explain the protective effect of physical activity on total and cancer mortality. In particular, exercise could lead to favourable effects on factors that contribute to cancer progression, including inflammation, immune function, oxidative stress, and metabolic hormones [14, 15].

Currently, research focused on the implementation and benefits of physical activity in later stages and metastatic cancer populations is significantly fewer than in patients with early-stage cancer, probably due to perceptions of physical disability and limitations in the former population. However, not all patients with metastatic or advanced cancer fall within the palliative or end-of-life cancer spectrum [16]. More than half of these patients are highly functional, with less than one impairment in activities of daily living and having good self-perceived quality of life until their last month of life [17]. Thus, introducing physical activity appears to be an appropriate intervention in this population. Three recently published reviews demonstrated that exercise interventions improved physical function, quality of life, and sleep quality, as well as reduced fatigue in patients with advanced cancer [18–21]. However, the effects of exercise on survival in this population remain inadequately understood due to the limited publications in survival data of advanced cancer patients, as well as being confined to reviews of longitudinal observational studies [12].

To the best of our knowledge, no systematic review or meta-analysis has been performed previously to investigate the effect of physical activity on survival in advanced cancer populations, encompassing both randomised control trials (RCTs) and non-randomised studies. This review, therefore, aimed to systematically evaluate and synthesize the effects of post-diagnosis physical activity on overall mortality in advanced cancer patients from all available non-randomised studies and RCTs, as well as appraise the methodological quality of the included studies.

## Methods

### Search strategy

This meta-analysis was conducted in accordance with the Preferred Reporting Items for Systematic Reviews and Meta-Analyses (PRISMA) guidelines [22]. A comprehensive literature search was conducted using the following databases: PubMed, EMBASE, CINAHL, PsycINFO, The Cochrane Central Register of Controlled Trials (CENTRAL), and SPORTDiscus, from the inception of the databases up to 3 February 2021. The following keywords “advanced”, “cancer”, “exercise”, and “survival”, and their medical subject headings or equivalent and text word terms were used as search terms. For an example of search strategy in PubMed, see Additional file 1. A manual review of the reference lists of the previously published meta-analyses and systematic reviews as well as from the selected articles was performed to identify potentially relevant articles.

### Eligibility criteria

Eligibility was determined using the following criteria: (1) peer-reviewed articles or published abstracts in English; (2) RCTs or non-RCTs (i.e., single-arm trial, cohort study, case-control study, cross-sectional study, and observational study); (3) presenting results on adult (aged > 18 years) cancer patients; (4) included the assessment of engagement in physical activity or physical activity as the intervention or a component of intervention; (5) investigating one or more survival outcomes; and (6) included ≥80% participants diagnosed as having advanced cancer or in studies in which separate analysis of advanced cancer is reported. Editorials, letters, comments, case reports, conference letters, qualitative research studies, systematic reviews, and meta-analyses were excluded.

The outcome of interest in this study was survival, measured at the end of the follow-up period through outcome measurements including survival probability, disease-free survival, cancer-specific mortality, and overall mortality. These outcomes were expressed as hazard ratios (HRs) or relative risks. The survival outcome measures that were commonly reported by the included studies were selected in this meta-analysis.

### Study selection

The selection was initially based on information in the title and abstract, with the entire manuscript examined if the initial information was inconclusive. Two reviewers (N.T. and S.L.C.) independently examined the search results, and disagreements were resolved by discussions to reach consensus. When more than one article reported on the same study, the article with the larger sample size or longer survival follow-up period was selected.

### Data extraction and quality assessment

Data extraction for all relevant studies was independently performed by two reviewers (N.T. and S.L.C.). All information regarding study characteristics (author, year, study design, year of publication), participant characteristics, physical activity characteristics and measures, survival outcome, follow-up duration, and summary of findings, were recorded using a predesigned data-extraction form. When insufficient data or unclear presentations were found in the articles, the corresponding authors were contacted for clarification.

The methodological quality of RCT was independently assessed by the two reviewers using the Cochrane Risk of Bias tool 2.0 (RoB 2) [23]. RoB 2 assessed five domains of bias: (1) bias arising from the randomisation process, (2) bias due to deviations from intended interventions, (3) bias due to missing outcome data, (4) bias in measurement of the outcome, and (5) bias in selection of the reported results. Studies were considered to have “low” or “high” risk-of-bias or “some concerns” in the overall risk-of-bias judgment.

On the other hand, the quality of observational studies was assessed using the Newcastle-Ottawa Quality Assessment Scale (NOS) on three domains: selection of exposed and unexposed cohorts (representativeness of the exposed cohort, selection of the unexposed cohort, ascertainment of exposure, and demonstration of absence of outcome at the beginning of studies), comparability of exposed and unexposed cohorts (analysis appropriately adjusted for potential confounding factors, including the most important and additional factors), and outcome ascertainment (adequacy of outcome assessment, length of follow-up, and adequacy of follow-up). A total score of 7 or more denotes high-quality studies [24]. The NOS is a comprehensive validated tool used to evaluate the quality of non-RCTs in meta-analysis [24].

### Statistical analyses

The pooled estimate for the association of physical activity with the outcome of interest was evaluated by combining study-specific HRs and 95% confidence intervals with random-effects meta-analysis models using log-transformed HR (lnHR). In studies where HR and its variance were not presented, the log HR from the included studies’ survival curves was estimated, obtaining survival results for both groups at time points along the survival curves to estimate HR and 95% confidence intervals between the physically active group and the control group. This approach was conducted as proposed by Parmar et al. [25]. Heterogeneity was investigated in each analysis using I^2^ values, which describe the percentage of variation across studies due to heterogeneity. Meta-regression was performed to further evaluate heterogeneity in terms of study characteristics. Moreover, we used separate analysis models for the study types (RCT and non-RCT). We performed sensitivity analysis using leave-one-out analysis to test whether individual studies disproportionately influenced the results. We used a trim-and-fill approach and funnel plots to investigate possible publication bias. All analyses of pooled effectiveness were conducted using STATA version 16.

## Results

### Study selection

**Fig. 1:**
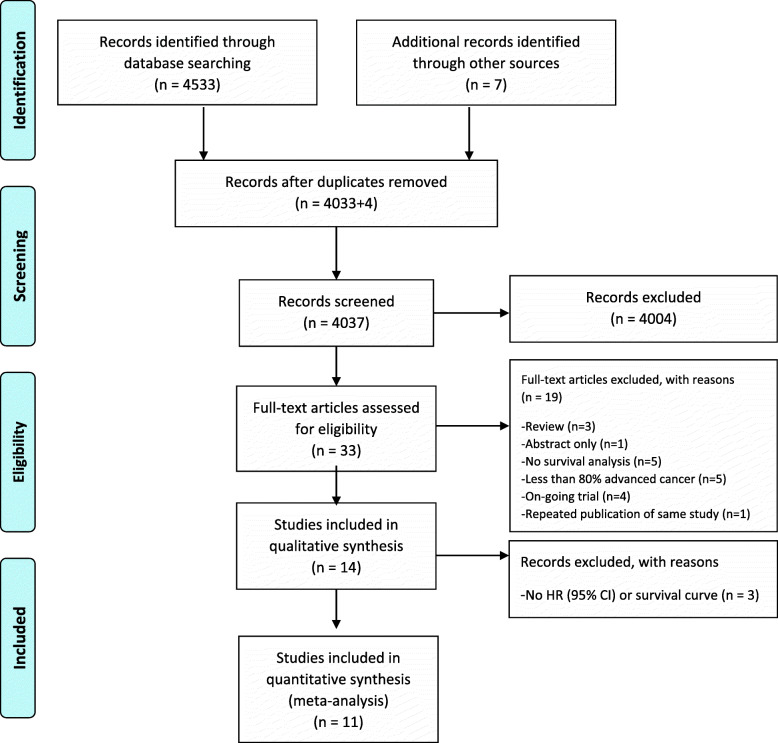
PRISMA Flow Diagram of Literature Search and Selection Process

The initial search of the specified electronic databases generated a total of 4533 studies, of which 4033 were deemed potentially relevant after removal of duplicates. An additional search of the reference lists returned seven potentially relevant articles, three of which were excluded, leaving 33 eligible studies for full-text review. A total of 14 studies that matched the inclusion criteria were included in the qualitative synthesis, with 11 of the 14 studies included in the meta-analysis due to the absence of HRs or survival curves (Fig. 1).

### Characteristics of the studies

Tables 1 and 2 summarise the characteristics of the 14 studies. Of the 14 included studies, six were RCTs [26–31] and eight were non-RCTs (one single-arm trial and seven cohort studies) [32–39] . The 14 included studies involved 3011 participants, with sample sizes ranging from 31 to 1218. The methodological quality of the studies is presented in Tables 3 and 4. The majority (5/6) of the included RCTs had a high overall risk of bias. The most common reasons for high risk of bias were the absence of blinding, low adherence to intervention, and inappropriate analyses used to estimate the effect of intervention adherence. On the other hand, four non-RCTs (4/8) scored 7 or greater, indicating a high quality. The most common reasons for reduction on the scale were the use of self-administered physical activity assessment tools, which have risks of overestimating the levels of physical activity compared to objective measures [40] and having a non-representative sample in the NOS scale, as studies recruited small sample sizes from a single clinic, limiting their generalizability.

**Table 1:** Characteristics of the randomized trials

Author/Year (in alphabetical order)	Number of subjects; Gender (female); Mean age, y	Study design	Setting	Cancer site (stage) Cancer treatment status	Physical activity intervention details (Duration, dose, intensity) Control group	Physical activity assessment tools	Survival outcome	Follow-up duration	Summary of results
Cheville et al. [26]	*N* = 66; IG: 33; CG: 33 Female: 47% Mean Age: 64.6	RCT	Single center: USA	Lung and colorectal cancer (IV); 51.5% undergoing treatment	IG: Home based incremental walking and strength training (moderate exertion) with bimonthly telephone calls Duration: 8 weeks CG: Usual care	Mean weekly step counts, REST sessions performed per week	Months to death from study enrollment	12 months	Survival did not differ significantly between the groups (HR: 0.92 for control group; *P* = 0.75).
Dhillon et al. [27]	*N* = 112; IG: 56; CG:55 Female: 45% Median age: 64	RCT	Multiple centers: Australia	Lung cancer (III-IV); Completed treatment (21.6%) or receiving anti-cancer treatment (78.4%)	IG: Physical activity program (supervised physical activity: 30–45 min and behavioral support session: 15–30 min + unsupervised home physical activity sessions + advice about resistance exercises + general health education materials) Duration: Once a week × 8 weeks CG: General health education materials only	Actigraph GT1M accelerometer (≥4 days), PA diary	Overall survival	Oct 2014 to July 2016	OS was not significantly different between groups, P _log-rank_ = 0.75; with median survival of 15.4 months (95% CI: 11.3, 24.1) EX and 13.2 months (95% CI: 11.1, 20.0) CG.
Oldervoll et al. [28]	*N* = 231; IG: 121; CG: 110 Female: 62.3% Mean age: 62.1	RCT	Multiple centers: Norway	Advanced and incurable cancer with heterogeneous cancer types; > 80% undergoing treatment	IG: Physical Exercise Intervention (Warm up, circuit training, stretching/ relaxation); 50–60 min Duration: Twice per week, 8 weeks CG: Usual care	One question about physical activity during leisure time over the past year	Overall survival	Median: 12.3 months	The unadjusted hazard ratio for survival (IG versus CG) was 1.24 (95% CI, 0.90–1.70; *p* = .18). After adjustment for age, gender, and KPS score, the hazard ratio was 1.19 (95% CI, 0.86–1.63; *P* = .30).
Rief et al. [29]	*N* = 60; IG: 30; CG: 30 Female: 45%; Mean age: 62.7	RCT	Single center: Germany	Cancer with spinal bone metastasis; Undergoing radiotherapy	IG: Resistance training for 2 weeks (30 mins) + home practice (3 times a week till 6 months) Duration: Monday to Fri, 2 weeks CG: Passive physical therapy	NR	Overall survival, Progression free survival	Median: 10 months	Overall survival after 12 and 24 months was 80 and 63% in IG, and 70 and 57% in CG respectively (*P* = 0.688).
Solheim et al. [30]	*N* = 46; IG: 25; CG: 21 Female: 56.5% Median age: 61	RCT (feasibility trial)	2 centers: Norway; 1 center: UK	Lung cancer (III/IV) or inoperable pancreatic cancer; Scheduled to start chemotherapy	IG: Home-based aerobic (30 mins; twice a week) and resistance training (20 mins; 3 times weekly) + nutritional counselling (30mins ×1 at baseline) + telephone follow-up Duration: 1–2 times a week, 6 weeks CG: Usual care	ActivPAL Steps (number of steps)	Overall survival	NR	The median (SD) survival in treatment arm was 10 (7) months and 8 (10) months in control arm (*P* = 0.57).
Uster et al. [31]	*N* = 58; IG: 29; CG: 29 Female: 31.0% Mean age: 63.0	RCT	Single center: Switzerland	Metastatic or locally advanced tumors of GI or lung tract cancer	IG: Nutritional counselling (min 3 sessions) + Physical exercise program (warm-up exercises, strength and balance training exercise; 60 mins) Duration: Twice a week, 3 months CG: Usual care	NR	Survival probability	3 months	The survival probability estimates at 3 months were 1.00 for the patients taking part in the intervention and 0.82 for those in the control group with no significant difference in survival rate (*P* = 0.25).

*Abbreviations*: *CI* confidence interval, *CG* control group, *HR* hazard ratio, *IG* intervention group, *KPS* Karnofsky Performance Status, *OS* overall survival, *PA* physical activity, *RCT* randomized controlled trials, *SD* standard deviation

**Table 2:** Characteristics of non-randomized trials

Author/Year (in alphabetical order)	Number of subjects Gender (female) Mean age, y	Study design	Setting	Cancer site (stage) Cancer treatment status	Physical activity type	Physical activity assessment tools	Survival outcome	Follow-up duration (years)	Confounding variables adjusted	Summary of results
Chiarotto et al. [32]	*N* = 35 Female: NR Mean age: 66.2	Pre-post, single arm	Single center: Canada	Incurable metastatic malignancy colorectal cancer; Undergoing chemotherapy	Supervised strength and aerobic exercise program + home practice everyday Duration: Once per week, 75 mins	NR	Overall survival (OS)	Till death	NR	Participation in the exercise pilot was not associated with any difference in survival (HR = 0.98, 95% CI 0.32–2.97).
Delrieu et al. [33]	*N* = 833 Female: 100% Mean age: 57.8	Prospective cohort	Multiple centers: France	Metastatic breast cancer; 52.6% undergoing chemotherapy	The average amount of time (in hours) spent weekly doing light and heavy household, moderate and vigorous recreational activity.	MET per minute and per week	Overall survival	Till death	Age at metastatic diagnosis, BMI ECOG, Performance Status, smokers, education, number of metastatic sites, adjuvant chemotherapy, metastatic at diagnosis and tumor type (Luminal-like, HER2+, Triple Negative).	After adjustment for multiple covariates and imputations on missing data, moderate and vigorous physical activity levels were not statistically significantly associated with longer survival in the whole population as compared to light physical activity (HR 0.95, 95% CI 0.70–1.29).
Guercio et al. [34]	*N* = 1218; Female: 41.1% Median age: 59.4	Prospective cohort	Multiple centers: USA and Canada	Advanced/ metastatic colorectal cancer; Within 1 month after chemotherapy initiation;	“During the past 2 months, what was your average time per week spent at each of the following recreational activities?” regarding nine leisure-time activities, as well as normal walking pace and number of stair flights per day.	Total MET hours per week	Overall survival, Progression-free survival	Median: 6.18 years	Age, sex, ECOG performance status, planned chemotherapy, prior adjuvant chemotherapy, prior radiation therapy, assigned treatment arm, BMI, primary tumour location, KRAS tumor status	Compared with individuals with less than 3 MET hours per week, individuals with 18 or more MET hours per week experienced a fully adjusted hazard ratio for OS of 0.85 (95% CI, 0.71 to 1.02; PTrend = .06).
Jones et al. [35]	*N* = 118 Female: 40% Mean age: 61	Prospective study	Single center: USA	Lung cancer (IIIB, IV or recurrent metastatic); 67% undergoing treatment	Average weekly exercise and duration since their primary adjuvant treatment consultation (MET)	Self-reported exercise behavior (Leisure score index by GLTEQ)	Overall survival	Median: 26.6 months	Age, gender, ECOG Performance status	Compared with patients reporting < 9 MET-hrs wk. − 1, the adjusted HR for mortality was 0.67 (95% CI, 0.31–1.48) for patients reporting ≥9 MET-hrs wk. − 1.
Lowe et al. [36]	*N* = 31 Female: 58% Mean age: 63.5	Cross-sectional	Single center: Canada	Progressive, incurable, cancer with brain metastatic; undergoing palliative whole brain radiotherapy	1. Supine or sitting position 2. Standing position 3. Stepping 4. Estimated energy expenditure 5. Number of steps over a 24-h period	ActivPAL accelerometer (for up to 7 days)	Overall survival	806 days	NR	No significant differences in median survival within the activity categories (standing position and supine or sitting position).
Ohri et al. [37]	*N* = 50 Female: 40% Mean age: 66	Prospective study	Single center: USA	Locally advanced lung cancer (> 80% stage III & IV); Scheduled for concurrent chemoradiation therapy	Daily step count (Inactive: 0–9000; Active: > 9000)	Wearable device for a median of 17 days (IQR: 12–20)	Overall survival; Progression-free survival	Median: 17.2 months	ECOG performance status	There was a trend suggesting an association between baseline activity level and OS (adjusted HR = 2.86) for inactive subjects; *P* = 0.62).
Palesh et al. [38]	*N* = 103 Female: 100% Mean age: 53.8	Prospective study	Multiple centers: USA	Breast cancer (Stage IV); 94% undergoing treatment	Amount of time spend engaged in various types of PA (METs/day)	Seven-Day Physical Activity Recall questionnaire	Overall survival	Mean: 60.43 months	Age, marital status, ER status, treatment received, metastatic disease spread [dominant site], depression, cortisol levels	The effect of physical activity as measured by METs on overall survival remained significant even after controlling for baseline prognostic factors (age, marital status, ER status, treatments received, metastatic disease spread [dominant site], depression, and cortisol levels) (HR: 0.91, CI: 0.84–0.99, *P* < .05).
Ruiz et al. [39] (abstract)	N = 50 Female: 21% Mean age: 68.5	Pilot prospective cohort	Multiple centers: USA	Newly diagnosed advanced lung cancer (stage IV); Scheduled for treatment	Low self-reported PA (< 383Kcals/week for men; < 270 Kcals/week for women)	Short Version of the Minnesota Leisure Time Activity Questionnaire	Overall survival	NR	Baseline hemoglobin, KPS, BMI	In multivariate analyses, low physical activity (HR 2.2, 95% CI: 1.2, 4.3) was independently associated with shorted survival.

*Abbreviations*: *BMI* body mass index, *CI* confidence interval, *ECOG* Eastern Cooperative Oncology Group, *ER* estrogen receptor, *HR* hazard ratio, *IQR* interquartile range, *KPS* Karnofsky Performance Status, *MET* metabolic equivalent, *NR* not reported, *OS* overall survival, *PA* physical activity

**Table 3:** Methodological quality of randomized trials

Author/ Year (in alphabetical order)	Randomization process	Deviation form intended intervention	Missing outcome data	Measurement of outcome	Selection of the reported result	Overall risk of bias
Cheville et al. [26]	L	L	L	L	S	S
Dhillon et al. [27]	L	H	L	L	L	H
Oldervoll et al. [28]	S	H	L	L	L	H
Rief et al. [29]	S	H	L	L	L	H
Solheim et al. [30]	L	H	L	L	L	H
Uster et al. [31]	L	H	L	L	S	H

*Note*: *H* High risk of bias, *L* Low risk of bias, *S* Some concerns

**Table 4:** Methodological quality of non-randomized trials

Author/ Year (in alphabetical order)	Selection	Comparability	Outcome	Total
Chiarotto et al. [32]	2	0	2	4
Delrieu et al. [33]	3	1	3	7
Lowe et al. [36]	3	0	3	6
Guercio et al. [34]	4	2	2	8
Ohri et al. [37]	3	1	1	5
Palesh et al. [38]	2	2	3	7
Jones et al. [35]	2	2	3	7
Ruiz et al. [39]	2	1	1	4

### Participant characteristics

Information about the participants are also shown in Tables 1 and 2. The mean age ranged from 53.8 to 68.5 years. Reporting of advanced stages of disease varied, with six studies defining the sample as advanced cancer by stage (IIIB/IV) [26, 27, 30, 35, 38, 39]. Four studies described the sample as having advanced cancer patients [28, 31, 34, 37], three studies characterized the sample as advanced based on their described pathologies [29, 32, 33], and one study by the palliative care treatment the patients received [36]. Six of the studies focused on multiple cancer sites [26, 28–31, 36], four on lung cancer [27, 35, 37, 39], two on colorectal cancer [32, 34], and breast cancer [33, 38]. Participants in nine of the studies were undergoing cancer treatment, were scheduled for treatment in three of the studies, and completed treatment in one of the studies, whereas one study did not specify treatment conditions.

### Physical activity type and assessment

All studies examined post-diagnosis physical activity. Among the six RCTs and one single-arm trial, six were a combination of aerobic and resistance training [26–28, 30–32], three of which consisted of an additional component of behavioural support or nutritional counselling [27, 30, 31]. The remaining one was resistance training alone [29]. Among the remaining seven cohort studies, the majority (5/7) of studies used self-administered questionnaires or questions [33–35, 38, 39] whereas the remaining two used accelerometers [36, 37]. The amount of physical activity in four studies was calculated by the metabolic equivalent (MET) [33–35, 38], one each by hours of standing, supine or sitting position [36], number of daily step count [37] and kilocalories per week [39].

### Survival outcome and assessment

All six RCTs and one single-arm trial compared survival outcomes between the intervention group and the control group or the non-participation control (Table 1). The associations between different levels of physical activities and survival outcomes were examined in the remaining seven cohort studies (Table 2). The median follow-up duration ranged from 8 months to 6.18 years.

### Description of the study results: summary of outcomes

The survival outcome between the intervention and control groups was not significant in all RCTs and the single-arm trial. Two out of the seven cohort studies demonstrated significant results between different levels of physical activities and survival outcomes, whereas the other four studies revealed insignificant results.

### Meta-analysis of studies: physical activity and cancer survival

**Fig. 2:**
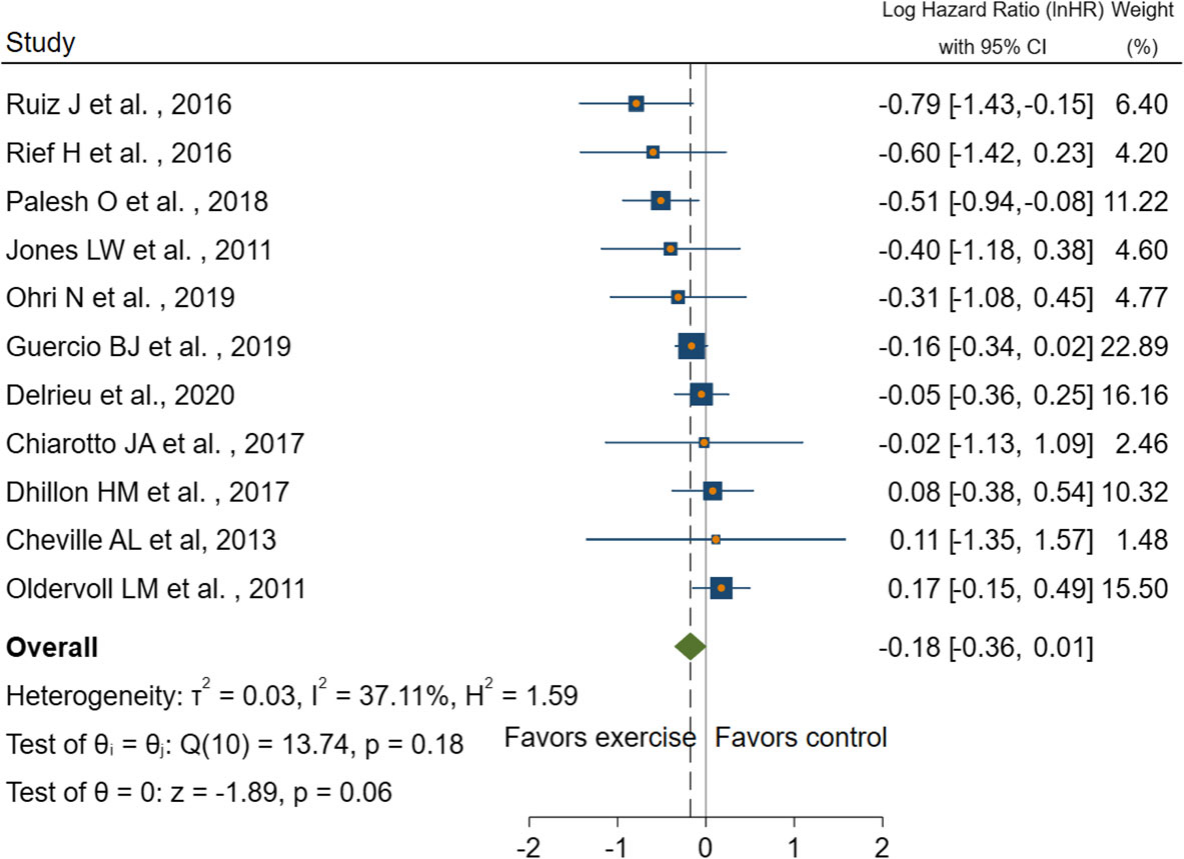
Forest plot for the association between PA and overall survival in advanced cancer patients. InHR with 95% CI log hazard ratios with 95% confidence intervals, lower scores in InHR favour intervention.Abbreviations. InHR: Log transformed hazard ratio; PA: Physical activity

**Fig. 3:**
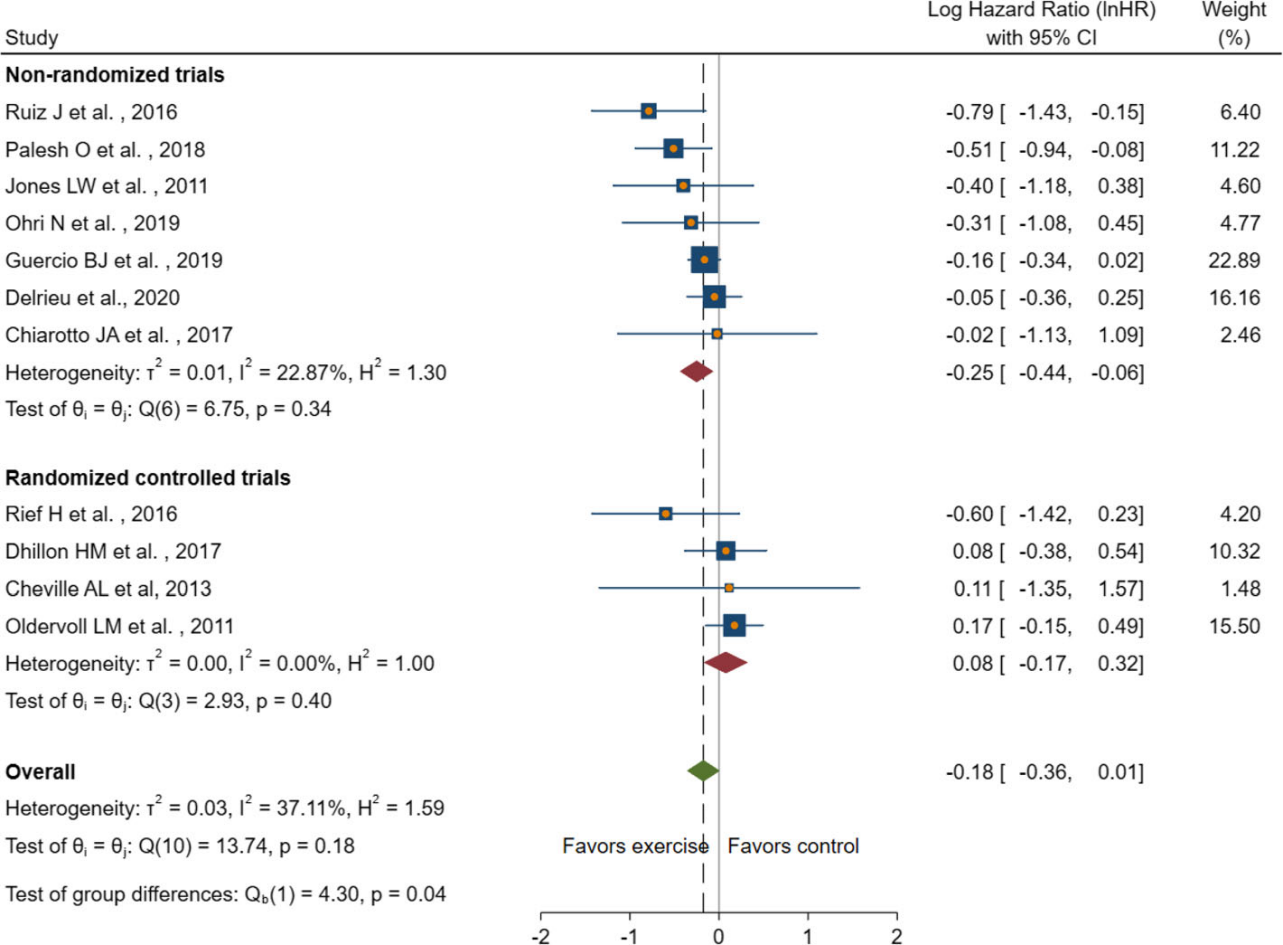
Forest plot for the association between PA and overall survival, as separated by study types. InHR with 95% CI log hazard ratios with 95% confidence intervals, lower scores in InHR favour intervention.Abbreviations. InHR: Log transformed hazard ratio; PA: Physical activity

**Fig. 4:**
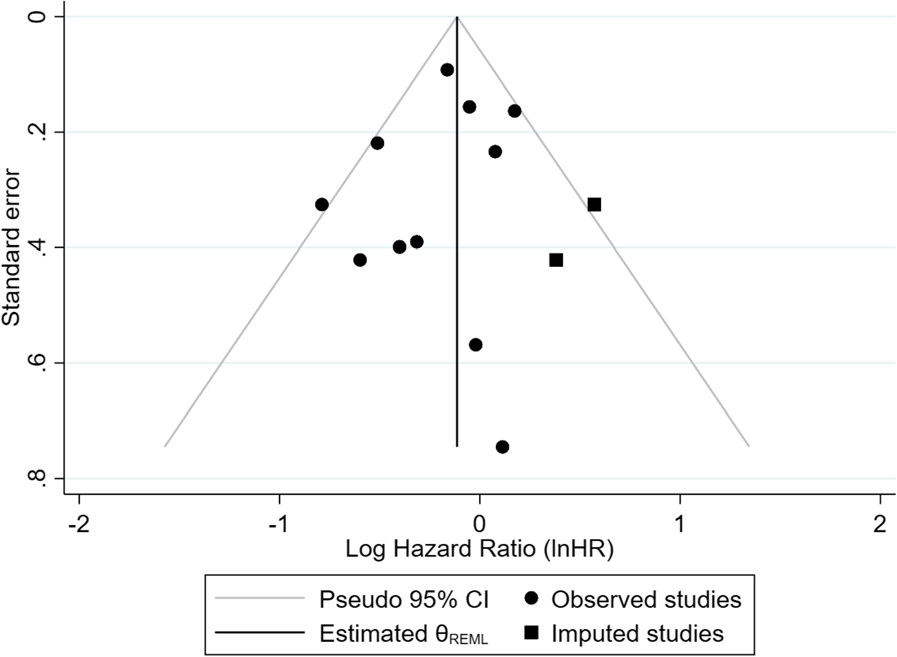
Funnel plot of all included studies. The circles indicate studies, and the triangle indicates added hypothetical studies from the trim-and-fill approach

Four RCTs and seven non-randomised trials (one single-arm trial and six cohort studies) were included in the meta-analysis. Figure 2 presents the results of the meta-analysis of physical activity and overall survival among advanced cancer patients. Compared with the reference group (lower-level physical activity or control group), higher-level physical activity was not significantly associated with a lower risk of earlier mortality in advanced cancer patients (InHR = − 0.18, 95% CI, − 0.36 to 0.01). Moreover, heterogeneity was moderate (I^2^ = 37.11%). A separate meta-analysis showed that study types played an effect on the heterogeneity of studies. For non-randomised trials, a higher level of physical activity was significantly associated with a reduced risk of mortality (InHR = − 0.25, 95% CI, − 0.44 to − 0.06, I^2^ = 22.87%) (Fig. 3). For randomised trials, participation in the intervention group was not significantly associated with a lower risk of mortality, compared with the control group (InHR = 0.08, 95%CI, − 0.17 to 0.32, I^2^ = 0.00%) (Fig. 3). The trim-and-fill analysis imputed two studies to increase symmetry in the funnel plot for all studies. The effect size from the observed and imputed studies did not change the significance of the meta-analysis with all included studies (InHR = − 0.11, 95%CI, − 0.31 to 0.08) (Fig. 4). The leave-one-out analysis was conducted among subgroups of studies (RCTs and non-randomised trials), and no change in significance was found when removing individual studies.

## Discussion

### Summary of evidence

To the best of our knowledge, this is the first meta-analysis examining the association between physical activity and overall survival in advanced cancer patients, comprising both randomised and non-randomised trials. Based on the 11 included studies, our comprehensive meta-analysis indicated with moderate heterogeneity that post-diagnosis physical activity had no effect on the overall survival among advanced cancer patients. However, heterogeneity was reduced to low levels when the analysis was separated by study type (randomised and non-randomised trials). Notably, a higher level of physical activity significantly reduced overall mortality in non-randomised trials, whereas the result remained insignificant for randomised trials.

Interestingly, significant associations between physical activity and survival were shown among non-randomised trials when the analysis was separated from RCTs. The discrepancies in the overall results in RCTs and non-RCTs may be attributed to the relatively shorter follow-up time (end-point range: 12–35 months) in RCTs than in non-RCTs (median range: 8–74 months). Hence, the survival data collected in RCTs might not be as complete as those in non-randomised trials to reflect the impact of physical activity on survival. In addition, RCT participants may have only started exercising after participating in the study, whereas for cohort studies, participants may have adopted the habit of exercise for a period of time before participating in the study. Therefore, the absence of significant findings in RCTs may reflect that it is too late for advanced cancer patients to start exercising to exert favourable effects on survival. All included RCTs did not exclude those with a history of exercise, and that may lead to contamination in the control group and minimal difference in physical activity between the two groups. Furthermore, the insignificant results yielded by comparing the overall survival between the intervention and control groups in the included RCTs might be attributed to the adherence to exercises. The adherence rate of intervention groups to the protocol of the physical activity intervention ranged from 69 to 76.9% [26–28], with one study not reporting adherence [29]. The amount of physical activity engaged in by the participants in the intervention group after the intervention period as well as the amount of physical activity engaged in by the control group at baseline were not reported in the included studies. Hence, it is possible that the intervention did not elicit a substantial difference in the amount of physical activity engaged in by the different groups. This finding is supported by one included study revealing minimal differences in the physical activity levels between groups during the study period, which might explain the lack of differences in outcomes [27]. Future RCTs should record and report the total amount of physical activity engaged in by both intervention and control groups to allow a more accurate comparison. In addition, three of the four included studies did not explicitly report HRs with variance; instead, the log HRs were estimated by the reviewers from the published survival curves. It should be noted that the results of the estimated log HRs might be subject to underestimation of the true impact of physical activity on survival [25].

Nonetheless, the significant results in the included non-randomised trials should be considered with the potential for bias and confounding within observational studies. The causation is difficult to imply based on the associations reported by the non-randomised trials included in our meta-analysis. For example, patients with higher levels of physical activity could be explained by the fact that they have better physical functioning and less symptom burden, and thus they are more active and could potentially have a longer survival duration. This assumption is supported by the findings of two included non-RCTs studies that higher proportion of patients with better performance status were reported in more physically active ones [33, 34]. Other limitations for observational studies include the possible risk of residual confounding factors that make it difficult to elucidate the real relationship between physical activity and overall survival.

We observed a growing number of studies conducted on physical activity in cancer patients since 2010, and there is still a paucity of evidence for physical activity among advanced cancer patients that could allow comprehensive and separate analyses by primary cancer sites. However, the optimal dose, modality, and timing of physical activity for advanced cancer patients in order to maximise its beneficial effects on survival outcomes remain unclear. Future studies could specifically include only one group of advanced cancer populations in order to generate more specific results and physical activity recommendations. Furthermore, only the effect of conventional physical exercises, namely aerobic or resistance or a combination of both exercises, on survival outcomes were reported in existing studies. There were yet to have studies evaluating the effect of mind-body exercise on survival. Increasing research has been conducted on mind-body exercise, another modality of exercise that combines body movement and meditation, such as tai chi, qigong, and yoga. A recent meta-analysis demonstrated that mind-body exercise led to significant improvements in various aspects such as physical fitness, fatigue, sleep quality, psychological distress in cancer survivors [41]. Therefore, future studies could investigate the effect of mind-body exercise on survival in cancer patients. Only post-diagnosis physical activity levels were assessed in the included studies, none of the studies investigated the pre-diagnosis physical activity levels. This maybe a confounding factor as previous studies found that pre-diagnosis physical activity levels is associated with survival in other cancer patients [42, 43]. Future studies should examine the pre-diagnosis physical activity levels that might have an impact on survival. Although physical activity was not directly associated with a reduction in the risk of mortality, other beneficial effects of physical activity in advanced cancer patients cannot be underestimated. The favourable effects demonstrated in previous reviews included improvement in physical function, sleep quality, quality of life, and reduction of depression, fatigue, and pain as well as decreased psychological complaints [18–21].

### Clinical implications

No evidence of detrimental effects of physical activity due to intervention was reported in the included studies. It might be possible that among advanced cancer patients, it is too late to start exercising for it to have effects on survival. Although the findings suggested that physical activity may have no effect on the overall survival in advanced cancer populations, healthcare professionals should consider the wider evidence of the beneficial effects of physical activity on other health-related outcomes, namely physical function, sleep quality, quality of life, and psychological distress [18–21].

### Strength and limitations

The strengths of this meta-analysis include its inclusion of both randomized and non-randomized trials, and studies reporting HRs with variance or survival curves. Thus, the results were more comprehensive in reflecting the association between physical activity and survival in an advanced cancer population. However, our meta-analysis has several limitations that merit further consideration. First, the number of studies included was relatively small, which limited the use of meta-regression for various factors as well as dose-response analyses. However, we were able to use study types (RCT and non-RCT) to explain the heterogeneity, and the heterogeneity reduced to 0 and 22.87%, respectively. Second, information on physical activity in non-randomised studies was self-reported except in two studies which utilized accelerometers; thus, it was prone to potential recall errors that may have biased the results. Future epidemiologic studies could adopt objective measurements of physical activity. Third, the majority of the included randomised trials did not account for other confounding risk modifiers, such as contamination in the control group that might potentially affect the results. Fourth, only 2 out of 7 non-RCTs have reported the performance status separately for the physically active and inactive cohorts, which might be a potential reason accounting for the differences in survival. Lastly, the log HRs in the four included studies were estimated from the published survival curves, which are prone to underestimation of the impact.

## Conclusions

In conclusion, this meta-analysis of currently available evidence suggests that participation in physical activity interventions may have no effect on overall survival among advanced cancer patients. However, the conclusion should be interpreted with the consideration of results possibly differing when randomised and non-randomised trials are separated. A higher level of physical activity was significantly associated with a lower risk of mortality in non-randomised trials, whereas no significant effect on survival was shown in exercise groups compared to control groups in RCTs. Given the safe nature and wider beneficial effects of physical activity, a set of recommendations for physical activity could be developed to serve as a component of survivorship for advanced cancer populations. The insignificant results in RCTs may be attributed to the presence of confounding risk modifiers such as contamination. There is a need for adequately powered, randomised, controlled exercise interventions to carefully interpret the impact of physical activity on survival and other clinically relevant outcomes such as number of hospital admissions, days spent in hospital, and financial toxicity.

## Supplementary Information

**Additional file 1.** Search strategy for PubMed. List of keywords used for literature search in PubMed.

## Data Availability

The datasets generated and/or analysed during the current study are available in PubMed, https://pubmed.ncbi.nlm.nih.gov/, EMBASE, https://www.embase.com/, CINAHL, https://www.ebsco.com/products/research-databases/cinahl-complete, PsycINFO, https://search.proquest.com/psycinfo/advanced/index, The Cochrane Central Register of Controlled Trials (CENTRAL), https://www.cochranelibrary.com/, and SPORTDiscus, https://www.ebsco.com/products/research-databases/sportdiscus.

## Abbreviations

CI: Confidence interval
HR: Hazard ratio
InHR: Log transformed hazard ratio
MET: Metabolic Equivalent
NOS: Newcastle-Ottawa Quality Assessment Scale
PRISMA: Preferred Reporting Items for Systematic Reviews and Meta-analyses
RCT: Randomized control trial
RoB: Risk of Bias tool

## References

[1] Cancer Research UK. Worldwide cancer statistics. https://www.cancerresearchuk.org/health-professional/cancer-statistics/worldwide-cancer#heading-One. Assessed 20 Nov 2020.

[2] Buyse M, Burzykowski T, Carroll K, Michiels S, Sargent DJ, Miller LL (2007). Progression-free survival is a surrogate for survival in advanced colorectal cancer. J Clin Oncol.

[3] Davidoff AJ, Tang M, Seal B, Edelman MJ (2010). Chemotherapy and survival benefit in elderly patients with advanced non–small-cell lung cancer. J Clin Oncol.

[4] American Cancer Society. Cancer A-Z. https://www.cancer.org/cancer.html. Assessed 20 Nov 2020.

[5] Cheung WY, Le LW, Gagliese L, Zimmermann C (2011). Age and gender differences in symptom intensity and symptom clusters among patients with metastatic cancer. Support Care Cancer.

[6] Penedo FJ, Dahn JR (2005). Exercise and well-being: a review of mental and physical health benefits associated with physical activity. Curr Opin Psychiatry.

[7] Speck RM, Courneya KS, Mâsse LC, Duval S, Schmitz KH (2010). An update of controlled physical activity trials in cancer survivors: a systematic review and meta-analysis. J Cancer Surviv.

[8] Sun Q, Townsend MK, Okereke OI, Franco OH, Hu FB, Grodstein F (2010). Physical activity at midlife in relation to successful survival in women at age 70 years or older. Arch Intern Med.

[9] Leitzmann MF, Park Y, Blair A, Ballard-Barbash R, Mouw T, Hollenbeck AR (2007). Physical activity recommendations and decreased risk of mortality. Arch Intern Med.

[10] Löllgen H, Böckenhoff A, Knapp G (2009). Physical activity and all-cause mortality: an updated meta-analysis with different intensity categories. Int J Sports Med.

[11] Rockhill B, Willett WC, Manson JE, Leitzmann MF, Stampfer MJ, Hunter DJ (2001). Physical activity and mortality: a prospective study among women. Am J Public Health.

[12] Barbaric M, Brooks E, Moore L, Cheifetz O (2010). Effects of physical activity on cancer survival: a systematic review. Physiother Can.

[13] Friedenreich CM, Stone CR, Cheung WY, Hayes SC (2020). Physical activity and mortality in cancer survivors: a systematic review and meta-analysis. JNCI Cancer Spectrum.

[14] Betof AS, Dewhirst MW, Jones LW (2013). Effects and potential mechanisms of exercise training on cancer progression: a translational perspective. Brain Behav Immun.

[15] Ballard-Barbash R, Friedenreich CM, Courneya KS, Siddiqi SM, McTiernan A, Alfano CM (2012). Physical activity, biomarkers, and disease outcomes in cancer survivors: a systematic review. J Natl Cancer Inst.

[16] Beaton R, Pagdin-Friesen W, Robertson C, Vigar C, Watson H, Harris SR (2009). Effects of exercise intervention on persons with metastatic cancer: a systematic review. Physiother Can.

[17] McCarthy EP, Phillips RS, Zhong Z, Drews RE, Lynn J (2000). Dying with cancer: patients’ function, symptoms, and care preferences as death approaches. J Am Geriatr Soc.

[18] Peddle‐McIntyre CJ, Singh F, Thomas R, Newton RU, Galvão DA, Cavalheri V. Exercise training for advanced lung cancer. Cochrane Database Syst Rev. 2019;(2)Art. No.:CD012685. 10.1002/14651858.CD012685.pub2. 10.1002/14651858.CD012685.pub2 PMC6371641 30741408

[19] Heywood R, McCarthy AL, Skinner TL (2018). Efficacy of exercise interventions in patients with advanced cancer: a systematic review. Arch Phys Med Rehabil.

[20] Dittus KL, Gramling RE, Ades PA (2017). Exercise interventions for individuals with advanced cancer: a systematic review. Prev Med.

[21] Takemura N, Cheung DST, Smith R, Deng W, Ho KY, Lin J, et al. Effectiveness of aerobic exercise and mind-body exercise in cancer patients with poor sleep quality: a systematic review and meta-analysis of randomized controlled trials. Sleep Med Rev. 2020;53:101334. 10.1016/j.smrv.2020.101334 32505970

[22] Moher D, Liberati A, Tetzlaff J, Altman DG, Group P (2009). Preferred reporting items for systematic reviews and meta-analyses: the PRISMA statement. PLoS Med.

[23] Sterne JA, Savović J, Page MJ, Elbers RG, Blencowe NS, Boutron I, et al. RoB 2: a revised tool for assessing risk of bias in randomised trials. BMJ. 2019;366:l4898. 10.1136/bmj.l4898 31462531

[24] Wells GASB, O'Connell D, Peterson J, Welch V, Losos M (2001). The Newcastle-Ottawa scale (NOS) for assessing the quality of nonrandomized studies in meta-analyses.

[25] Parmar MK, Torri V, Stewart L (1998). Extracting summary statistics to perform meta-analyses of the published literature for survival endpoints. Stat Med.

[26] Cheville AL, Kollasch J, Vandenberg J, Shen T, Grothey A, Gamble G (2013). A home-based exercise program to improve function, fatigue, and sleep quality in patients with stage IV lung and colorectal cancer: a randomized controlled trial. J Pain Symptom Manag.

[27] Dhillon H, Bell ML, van der Ploeg H, Turner J, Kabourakis M, Spencer L (2017). Impact of physical activity on fatigue and quality of life in people with advanced lung cancer: a randomized controlled trial. Ann Oncol.

[28] Oldervoll LM, Loge JH, Lydersen S, Paltiel H, Asp MB, Nygaard UV (2011). Physical exercise for cancer patients with advanced disease: a randomized controlled trial. Oncologist.

[29] Rief H, Bruckner T, Schlampp I, Bostel T, Welzel T, Debus J (2016). Resistance training concomitant to radiotherapy of spinal bone metastases–survival and prognostic factors of a randomized trial. Radiat Oncol.

[30] Solheim TS, Laird BJ, Balstad TR, Stene GB, Bye A, Johns N (2017). A randomized phase II feasibility trial of a multimodal intervention for the management of cachexia in lung and pancreatic cancer. J Cachexia Sarcopenia Muscle.

[31] Uster A, Ruehlin M, Mey S, Gisi D, Knols R, Imoberdorf R (2018). Effects of nutrition and physical exercise intervention in palliative cancer patients: a randomized controlled trial. Clin Nutr.

[32] Chiarotto JA, Akbarali R, Bellotti L, Dranitsaris G (2017). A structured group exercise program for patients with metastatic cancer receiving chemotherapy and CTNNB1 (β-catenin) as a biomarker of exercise efficacy. Cancer Manag Res.

[33] Delrieu L, Jacquet E, Segura-Ferlay C, Blanc E, Febvey-Combes O, Friedenreich C (2020). Analysis of the StoRM cohort reveals physical activity to be associated with survival in metastatic breast cancer. Sci Rep.

[34] Guercio BJ, Zhang S, Ou F-S, Venook AP, Niedzwiecki D, Lenz H-J (2019). Associations of physical activity with survival and progression in metastatic colorectal cancer: results from Cancer and Leukemia Group B (Alliance)/SWOG 80405. J Clin Oncol.

[35] Jones LW, Hornsby WE, Goetzinger A, Forbes LM, Sherrard EL, Quist M (2012). Prognostic significance of functional capacity and exercise behavior in patients with metastatic non-small cell lung cancer. Lung Cancer.

[36] Lowe SS, Danielson B, Beaumont C, Watanabe SM, Baracos VE, Courneya KS (2014). Associations between objectively measured physical activity and quality of life in cancer patients with brain metastases. J Pain Symptom Manag.

[37] Ohri N, Halmos B, Bodner WR, Cheng H, Guha C, Kalnicki S (2019). Daily step counts: a new prognostic factor in locally advanced non-small cell lung cancer?. Int J Radiat Oncol Biol Phys.

[38] Palesh O, Kamen C, Sharp S, Golden A, Neri E, Spiegel D (2018). Physical activity and survival in women with advanced breast cancer. Cancer Nurs.

[39] Ruiz J, Miller AA, Tooze J, Petty WJ, Gajra A, Klepin HD (2016). Association of self-reported physical activity with survival among patients with advanced non-small cell lung cancer (NSCLC).

[40] Cerin E, Cain KL, Oyeyemi AL, Owen N, Conway TL, Cochrane T (2016). Correlates of agreement between accelerometry and self-reported physical activity. Med Sci Sports Exerc.

[41] Duan L, Xu Y, Li M. Effects of mind-body exercise in cancer survivors: a systematic review and meta-analysis. Evid Based Complement Alternat Med. 2020;2020:Article ID 7607161. 10.1155/2020/7607161. 10.1155/2020/7607161 PMC7487122 32952591

[42] Wu W, Guo F, Ye J, Li Y, Shi D, Fang D (2016). Pre-and post-diagnosis physical activity is associated with survival benefits of colorectal cancer patients: a systematic review and meta-analysis. Oncotarget..

[43] Schmidt ME, Chang-Claude J, Vrieling A, Seibold P, Heinz J, Obi N (2013). Association of pre-diagnosis physical activity with recurrence and mortality among women with breast cancer. Int J Cancer.

