# Supplementary material for: The effects of physical activity on overall survival among advanced cancer patients: a systematic review and meta-analysis
Source: BMC Cancer. 2021 Mar 7;21:242. doi: 10.1186/s12885-021-07988-1 (PMC7938536; doi:10.1186/s12885-021-07988-1)
Supplement: Supplementary file 1 — Additional file 1. Search strategy for PubMed. List of keywords used for literature search in PubMed. [file 12885_2021_7988_MOESM1_ESM.docx]

**Title:** The effects of physical activity on overall survival among advanced cancer patients: A systematic review and meta-analysis

**Authors:** Naomi TAKEMURA^a^, Siu Ling CHAN^a^, Robert SMITH^a^, Denise Shuk Ting CHEUNG^a^, Chia-Chin LIN^a,b,c^

**Additional file 1.**

(((advanced OR incurable OR metastat*) AND ((cancer* OR neoplasm* OR tumor* OR tumour OR malignan*))) AND (Exercise OR exercise tolerance OR exertion OR Pliability OR physical fitness OR Physical Education and Training OR physical endurance OR exercise therapy OR exercising OR physical condition* OR stamina OR motor activity OR exercise test OR Sports OR tai chi OR Tai ji OR yoga OR muscle stretching exercises OR range of motion articular OR plates OR qigong OR chi kung OR resistance training OR Strength* exercis* or isometric* exercis* or isotonic* exercis* or isokinetic* exercis* or aerobic* exercis* or endurance exercis* or weight* exercis* OR Strength* train* or isometric* train* or isotonic* train* or isokinetic* train* or aerobic* train* or endurance train* or weight* train* OR muscle strength* OR mind body therap* OR complementary therapies OR Water based exercise OR Ai Chi OR Halliwick OR hippotherapy OR Hydrotherapy OR balance exercise OR aquatic exercise)) AND (survival OR mortality OR prognosis)
